# Peripheral blood lymphocytes immunophenotyping predicts disease activity in clinically isolated syndrome patients

**DOI:** 10.1186/s12883-017-0915-1

**Published:** 2017-07-28

**Authors:** Helena Posová, Dana Horáková, Václav Čapek, Tomáš Uher, Zdenka Hrušková, Eva Havrdová

**Affiliations:** 1 Institute of Immunology and Microbiology, First Faculty of Medicine, Charles University and General University Hospital in Prague, Prague, Czech Republic; 2 Department of Neurology and Centre of Clinical Neuroscience First Faculty of Medicine, Charles University and General University Hospital in Prague, Prague, Czech Republic

**Keywords:** Clinically isolated syndrome, Multiple sclerosis, Lymphocyte subpopulations, Flow cytometry

## Abstract

**Background:**

Clinically isolated syndrome (CIS) represents first neurological symptoms suggestive of demyelinating lesion in the central nervous system (CNS). Currently, there are no sufficient immunological or genetic markers predicting relapse and disability progression, nor there is evidence of the efficacy of registered disease modifying treatments (DMTs), such as intramuscular interferon beta1a. The aim of the study is to evaluate immunological predictors of a relapse or disability progression.

**Methods:**

One hundred and eighty one patients with CIS were treated with interferon beta1a and followed over the period of 4 years. Lymphocyte subsets were analyzed by flow cytometry. A Kaplan-Meier estimator of survival probability was used to analyze prognosis. For statistical assessment only individual differences between baseline values and values at the time of relapse or confirmed disability progression were analysed.

**Results:**

Higher levels of B lymphocytes predicted relapse-free status. On the other hand, a decrease of the naïve subset of cells (CD45RA+ in CD4+) after 12, 24, and 36 months of follow-up were associated with an increased risk of confirmed disability progression. Conclusion: Our data suggest that the quantification of lymphocyte subsets in patients after the first demyelinating event suggestive of MS may be an important biomarker.

## Background

Clinically isolated syndrome (CIS) is the first manifestation of multiple sclerosis (MS), a chronic inflammatory autoimmune disease of the central nervous system (CNS) affecting over 2.5 million people worldwide. [1] After CIS, the delay in manifestation of a new relapse, which corresponds to the clinically definite MS (CDMS) according to McDonald’s criteria 2005 [2], varies from several months to more than 10 years and should be associated with the brain and spinal cord pathology at the time of a relapse [3]. In about a third of the cases, CIS patients will not experience a new relapse activity over long-term follow-up [4].

Different clinical and brain imaging predictors have been evaluated for their sensitivity and specificity to predict occurrence of a relapse during follow-up. In this context, a number of studies have shown that abnormal MRI findings are the most informative predictors of future disease activity [5, 6].

On the other hand, the role of cerebrospinal fluid (CSF) biomarkers for the prediction of a relapse remains to be explored. It is also unclear if MRI predictors together with CSF biomarkers may improve prediction of CDMS when applied in a single model. Apart from oligoclonal bands (OCBs), several markers in the CSF appear to be more specific for neuro-inflammatory and neuro-degenerative pathophysiological processes in MS, such as inflammation and immune dysfunction, or cell type, such as B cells [7]. Some of these markers have been shown to predict conversion to MS in patients with CIS, e.g. polyspecific intrathecal B-cell response of IgG antibodies against neurotropic viruses such as measles, rubella, and varicella zoster [8]. CSF IgG heavy-chain bias was detected in patients with CIS who converted to MS within 6 months of the CIS presentation [9], and increased CSF concentrations of B cell recruiting chemokine CXCL13 were shown to be a good predictor of conversion to MS in patients with CIS over 2 years of follow-up [10]. It has been suggested that Tau and neurofilaments, CSF markers of axonal damage, might be even more specific than MRI for predicting conversion of CIS to MS. [11] Shorter time to CDMS conversion was also associated with high concentrations of CSF chitinase 3-like 1, which is up-regulated during inflammation [12].

Nevertheless, up to date none of these CSF markers can be recommended for routine implementation in clinical practice, mainly because of methodological limitations, invasiveness of the spinal tap, absence of conclusive data from small studies or other technical problems. Therefore large multicentre studies are needed to confirm the importance of CSF biomarkers as a tool in the clinical practice [13]. CSF is the compartment in the closest proximity to CNS parenchyma and might reflect immunopathology in CNS. However, studies usually fail to provide longitudinal CSF data because repeated lumbar punctures are difficult to justify. Therefore, single CSF samples are only snapshots of immune response at the time of collection.

In this context, peripheral blood as a mirror of immune reaction in CNS is much easier to measure longitudinally. CD4+ T cells and later CD4 + Th1 cells are the most studied cell populations in MS [14] because of their potential role in the pathogenesis of the disease, as well as they are used to assess the effect of different therapies used in MS. More recently, regulatory subpopulations such as Th2 cells, regulatory CD4+ T cells and NK cells were studied for their relationship in disease prognosis [15] and radiologically confirmed MS activity. Changes in their effector populations were described [16]. The association of CD4 + CD45RO+ IL-17A+ cells to clinical and radiological disease activity was reported. [17, 18] Differences in naive CD4 T-cell biology, notably in TCR and TLR signalling pathways, identified patients with MS with more rapid conversion to secondary progression [19].

In CIS patients, the most intriguing finding was published by Vilar et al. [20], who showed the possibility of using an analysis of peripheral blood CD5 + CD19+ subsets as a predictive factor for CDMS conversion.

In our study, we followed-up patients with CIS who were treated with interferon beta, irrespective of the further disease course (patients with both subsequent relapse and no relapse were included) for at least 48 months. Peripheral blood immunophenotyping was used to find early changes in lymphocyte subsets, which could predict the development of relapse.

## Methods

### Study population

The study enrolled patients after first clinical event suggestive of MS within 4 months from the first clinical event. The inclusion criteria were:18–55 years of age, enrolled within 4 months from the clinical event, Expanded Disability Status Scale (EDSS) ≤3.5, presence of ≥2 T2-hyperintense lesions on diagnostic MRI, and presence of ≥2 oligoclonal bands in CSF obtained at the screening visit prior to steroid treatment. The exclusion criteria were: occurrence of a second relapse before the baseline visit, pregnancy and symptoms that could possibly be attributed to neurological diseases different from MS (e.g. neuromyelitis optica).

All patients included with CIS were treated with intravenous steroids (3–5 g of methylprednisolone) following screening and preceding baseline, and subsequently at baseline started the treatment with 30 mg of intramuscular IFNb-1a once a week. Two hundred and twenty CIS patients were enrolled in the study; analyses in this laboratory part of study were limited to 191 subjects with immunophenotyping flow cytometry data available. Clinical assessments (EDSS) and peripheral blood assessments were obtained at baseline, 6, 12, 24, 36 and 48 months. In the case of relapses, patients were evaluated within 4 days from the onset of new symptoms.

The study protocol was approved by the Medical Ethics Committee of the General University Hospital in Prague and First Faculty of Medicine, Charles University in Prague for grant MZ ČR NT 13108–4.

### Study outcomes

We used two clinical outcomes in this analysis – 1) development of a new clinical relapse, and 2) confirmed disability progression. A new clinical relapse was defined as patient-reported symptoms or objectively observed signs typical for an acute inflammatory demyelinating event in the central nervous system with duration of at least 24 h, in the absence of fever or infection. [21] Confirmed disability progression (CDP) was defined as an increase in EDSS by 1.0 point (if baseline EDSS >0) or 1.5 points (if baseline EDSS = 0) confirmed after 6 months [22].

### Immunophenotyping

Whole peripheral blood (PB) samples (2 ml/subject) were collected in ethylenediamine tetra-acetic acid (EDTA) test tubes. The following monoclonal antibodies from BD Biosciences (San Jose, CA, USA) were used: CD3 FITC, CD4 PE-Cy7, CD5 FITC, CD8 APC Cy7, CD19 PE, CD45RA FITC, HLA-DR PE Cy7, CD16 + 56 PE and CD45 PerCP. Whole PB samples were labelled with appropriate volumes of conjugated MoAb for 20 min at room temperature, and then lysed with 2 ml of lysis solution (FACS Lysis Solution, BD). Cells were washed twice and analyzed on a standard FACSCanto instrument (BD) with DIVA software (BD). All results were expressed as percentages of each subset out of total lymphocytes.

### Statistical methods

Percentages of lymphocyte populations do not have normal distribution and therefore, the parametric tests cannot be applied. Moreover, the results at the end of the study were influenced by different treatment modalities in study patients. For statistical assessment, only individual differences between baseline values and values at the time of relapse were used and compared with various indicators.

Predictive values of measured variables were investigated through receiver operating characteristic (ROC) curves. Based on these curves, thresholds which maximised the sum of sensitivity and specificity for each measured parameter were defined. A Kaplan-Meier estimator of survival probability and an asymptotic log-rank test were used to test differences between subsets of patients. For each lymphocyte population and the clinical outcome (relapse or CDP) three different indicators were considered: a) the population relative value, b) the population relative value change compared to baseline and c) the population relative value change compared to a measurement one year before.

For all indicators the following analyses were performed:

1. An ROC curve to predict the clinical outcome at one year was constructed and an optimal threshold was chosen.
2. At every time-point a cohort of patients was divided into two groups according to the chosen threshold and survival curves (Kaplan-Meier estimators) were constructed for each of these groups. Further, an asymptotic log-rank test was performed to test difference between these survivals probability curves (see Figures and Tables). *P*-values less than 0.05 were considered as statistically significant.

The ROC curves were constructed from all the time points at once. As a predicted outcome we considered a relapse or disease progression, respectively that appeared in (and only in) a year after the corresponding measurement. The events were analysed from the year end. The same threshold applied to measurements before baseline, at baseline, at 6 M, etc. Every time it divided patients into two groups. For those groups the survival probability curves were constructed and compared.

Analyses were conducted using R statistical package, version 3.1.2, R Core Team (2014).

## Results

### Clinical data

Of the 220 patients enrolled in the study, 191 had available clinical and laboratory follow-up data (181 till the end of study, not only till relapse or disability progression) and were included in the analysis. During the first year of follow-up, second disease relapse was observed in 55 patients, during the second year additional 25 patients relapsed, and 21 and 13 patients, respectively, relapsed during the third and the fourth year of the follow-up. At 48 months, 114 (59.6%) patients experienced the second clinical attack, 69 (36.1%) experienced the third clinical attack and 37 (19.3%) had confirmed disability progression (CDP).

### Laboratory data

Representation of different lymphocyte subpopulations in patients who did not experience a relapse was compared to patients who relapsed within the respective time period (6, 12, 24, 36 and 48 months). The same comparison was done for patients who showed CDP. Patients who underwent both events (relapse and CDP) were included in both subgroups. Table 1a, b shows the number of patients in each subgroup and median ± standard deviation of measured lymphocyte subpopulations. Absolute counts of lymphocyte subpopulations are shown in Table 2a, b.

**Table 1:** a, b Median ± standard deviation of lymphocyte subpopulations (in % of all lymphocytes) in different subgroups

a							
	n	T ly	CD4+	CD8+	B ly	NK cells	CD5+
all patients at first relapse	191	71.8 ± 7	44.6 ± 7	23 ± 6	11.1 ± 4	14 ± 7	72 ± 7
all patients at baseline (BL)^a^	191	71.5 ± 7	43 ± 8	24 ± 7	9.3 ± 4	16.1 ± 7	71 ± 9
all patients at 48 months (48)^b^	181	72.5 ± 7	45 ± 7	21.7 ± 6	15.3 ± 6	9.6 ± 5	73.9 ± 6
patients without relapse at BL	77	71.2 ± 7	42.3 ± 8	23.4 ± 7	9.3 ± 4	16.5 ± 8	71.2 ± 8
patients without relapse at 48^b^	77	73 ± 6	46.2 ± 6	22.1 ± 6	14.8 ± 5	9.45 ± 4	74.5 ± 5
patients with relapse at BL	114	71.7 ± 8	43.5 ± 7	24.5 ± 6	9.3 ± 4	15.4 ± 7	70.7 ± 10
patients with relapse at 48^b^	104	72.1 ± 8	45.9 ± 8	21.4 ± 6	15.8 ± 7	9.8 ± 5	73.6 ± 7.
patients without CDP at BL	154	71.3 ± 8	42.2 ± 8	24.5 ± 7	9.1 ± 4	17 ± 7	70.8 ± 9
patients without CDP at 48^b^	148	72.5 ± 7	45.7 ± 8	22.2 ± 6	15.3 ± 6	9.9 ± 5	73.6 ± 6
patients with CDP at BL	37	73.4 ± 6	47 ± 7	23.1 ± 6	10 ± 4	13 ± 6	72.8 ± 6
patients with CDP at 48^b^	33	74.6 ± 7	47.8 ± 7	20.5 ± 5	16.3 ± 6	8.4 ± 3	75.3 ± 6
b							
	n	DR+ in T ly	CD45RA+	CD45RA+ in CD4+	CD5+ in B ly	CD5+ B ly	
all patients at first relapse	191	10.8 ± 9	65.4 ± 11	58.8 ± 19	20 ± 16	2.1 ± 2	
all patients at baseline (BL)^a^	191	12.1 ± 11	65.2 ± 10	56.4 ± 20	18.1 ± 19	1.7 ± 2	
all patients at 48 months (48)^b^	181	12.2 ± 6	65.1 ± 8	44.8 ± 12	11.1 ± 7	1.7 ± 1	
patients without relapse at BL	77	12.9 ± 11	64.9 ± 9	57.5 ± 20	23.3 ± 18	1.9 ± 3	
patients without relapse at 48^b^	77	12.1 ± 6	64.3 ± 8	45.2 ± 12	11.4 ± 8	1.9 ± 2	
patients with relapse at BL	114	11.8 ± 10	63.4 ± 11	55.6 ± 21	18 ± 14	1.7 ± 2	
patients with relapse at 48^b^	104	12.8 ± 5	66.2 ± 8	44.8 ± 13	10 ± 6	1.7 ± 2	
patients without CDP at BL	154	12.5 ± 10	64.8 ± 10	54.9 ± 20	18.1 ± 20	1.7 ± 2	
patients without CDP at 48^b^	148	12.4 ± 6	66.1 ± 8	45.8 ± 13	11.6 ± 8	1.9 ± 1	
patients with CDP at BL	37	9.8 ± 9	62.4 ± 12	60.5 ± 23	16.7 ± 14	1.9 ± 2	
patients with CDP at 48^b^	33	11.1 ± 5	62.7 ± 9	40.7 ± 11	9.7 ± 5	1.4 ± 1	

Double positive populations (HLA-DR + CD3+, CD45RA + CD4+, CD5 + CD19+) are expressed as the percentage of the first mentioned subpopulation in the basic population. CD5+ B lymphocytes are expressed as the originally measured value (percentage of total lymphocytes) to enable comparison with previously published results. [20]

^a^The laboratory test were done before intravenous steroids treatment, interval between this examination and baseline was 2–3 months

^b^The results at 48 months of following should be influenced by treatment (natalizumab *n* = 18, fingolimod *n* = 3, copaxone *n* = 6)

T ly – T lymphocytes (CD3+), B ly – B lymphocytes (CD19+), NK cells (CD3-CD16 + 56+)

**Table 2:** a, b Median ± standard deviation of lymphocyte subpopulations (absolute count in 10^3^/ml) in different subgroups

a							
	n	T ly	CD4+	CD8+	B ly	NK cells	CD5+
all patients at first relapse	191	1.3 ± 0.4	0.8 ± 0.3	0.4 ± 0.2	0.2 ± 0.1	0.2 ± 0.1	1.3 ± 0.4
all patients at baseline (BL)^a^	191	1.3 ± 0.4	0.8 ± 0.3	0.5 ± 0.2	0.2 ± 0.1	0.3 ± 0.2	1.3 ± 0.4
all patients at 48 months (48)^b^	181	1.3 ± 0.5	0.8 ± 0.3	0.4 ± 0.2	0.3 ± 0.2	0.2 ± 0.1	1.3 ± 0.5
patients without relapse at BL	77	1.3 ± 0.3	0.8 ± 0.2	0.4 ± 0.2	0.2 ± 0.1	0.3 ± 0.2	1.3 ± 0.3
patients without relapse at 48^b^	77	1.3 ± 0.4	0.8 ± 0.2	0.4 ± 0.2	0.3 ± 0.1	0.2 ± 0.1	1.4 ± 0.4
patients with relapse at BL	114	1.3 ± 0.5	0.8 ± 0.3	0.5 ± 0.2	0.2 ± 0.1	0.3 ± 0.1	1.3 ± 0.5
patients with relapse at 48^b^	104	1.3 ± 0.5	0.8 ± 0.3	0.4 ± 0.2	0.2 ± 0.2	0.2 ± 0.1	1.3 ± 0.6
patients without CPD at BL	154	1.3 ± 0.4	0.8 ± 0.3	0.4 ± 0.2	0.2 ± 0.1	0.2 ± 0.1	1.3 ± 0.4
patients without CPD at 48^b^	148	1.3 ± 0.5	0.8 ± 0.3	0.4 ± 0.2	0.3 ± 0.2	0.2 ± 0.1	1.4 ± 0.5
patients with CPD at BL	37	1.2 ± 0.4	0.8 ± 0.2	0.3 ± 0.2	0.2 ± 0.1	0.2 ± 0.1	1.2 ± 0.4
patients with CPD at 48^b^	33	1.1 ± 0.5	0.8 ± 0.3	0.3 ± 0.2	0.3 ± 0.2	0.1 ± 0.1	1.2 ± 0.6
b							
	n	DR+ CD3+	CD45RA+	CD45RA+ CD4+	CD5+ B ly		
all patients at first relapse	191	0.1 ± 0.1	1.1 ± 0.4	0.5 ± 0.2	0.04 ± 0.05		
all patients at baseline (BL)^a^	191	0.2 ± 0.1	1.2 ± 0.4	0.5 ± 0.3	0.03 ± 0.05		
all patients at 48 months (48)^b^	181	0.2 ± 0.1	1.1 ± 0.5	0.4 ± 0.2	0.03 ± 0.03		
patients without relapse at BL	77	0.2 ± 0.1	1.2 ± 0.3	0.5 ± 0.2	0.03 ± 0.05		
patients without relapse at 48^b^	77	0.2 ± 0.1	1.1 ± 0.4	0.4 ± 0.2	0.03 ± 0.04		
patients with relapse at BL	114	0.2 ± 0.1	1.2 ± 0.5	0.5 ± 0.3	0.03 ± 0.04		
patients with relapse at 48^b^	104	0.1 ± 0.1	1.1 ± 0.6	0.4 ± 0.2	0.03 ± 0.03		
patients without CDP at BL	154	0.2 ± 0.1	1.2 ± 0.4	0.5 ± 0.3	0.04 ± 0.05		
patients without CPD at 48^b^	148	0.1 ± 0.1	1.1 ± 0.5	0.4 ± 0.2	0.03 ± 0.03		
patients with CPD at BL	37	0.1 ± 0.2	1 ± 0.4	0.4 ± 0.2	0.04 ± 0.04		
patients with CPD at 48^b^	33	0.1 ± 0.1	1 ± 0.6	0.3 ± 0.2	0.02 ± 0.03		

^a^The laboratory test were done before intravenous steroids treatment, interval between this examination and baseline was 2–3 months

^b^The results at 48 months of following should be influenced by treatment (natalizumab *n* = 18, fingolimod *n* = 3, copaxone *n* = 6)

T ly – T lymphocytes (CD3+), B ly – B lymphocytes (CD19+), NK cells (CD3-CD16 + 56+)

The results are shown for a better orientation and to complement the statistical analysis, but a few trends were observed in the results, the most noticeable ones were the increase in B lymphocytes and the decrease in NK cells. These trends were present in all subgroups, regardless of the clinical status (conversion to CDMS or no conversion). However, a profound decrease in B lymphocytes and an increase in NK cells was observed between the study entry and the baseline results and of at least 30 days prior to initiation of interferon therapy (of at least 30 days after steroid administration).

### Statistical analysis

**Fig. 1:**
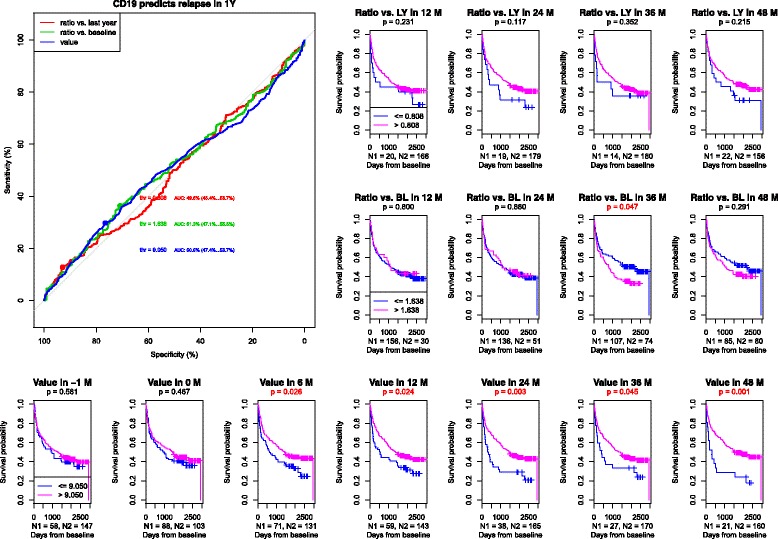
1) ROC curves of CD19+ B lymphocytes to predict relapse at one year. AUC for the population relative value = 0.505, AUC for the population relative value change compared to baseline = 0.513, AUC for the population relative value change compared to a measurement one year before = 0.495. 2) Survival curves and asymptotic log-rank tests for groups divided by the threshold

**Fig. 2:**
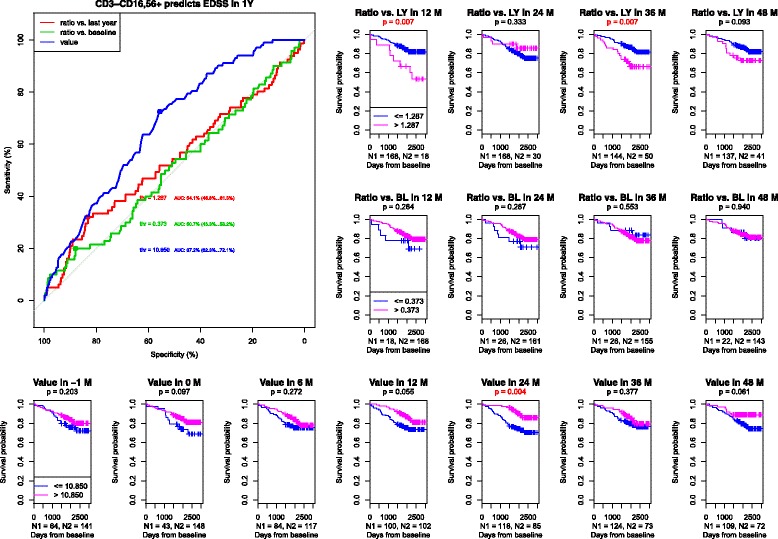
1) ROC curves of CD3-CD16 + 56+ NK cells to predict CDP at one year. AUC for the population relative value = 0.672, AUC for the population relative value change compared to baseline = 0.507, AUC for the population relative value change compared to a measurement one year before = 0.541. 2) Survival curves and asymptotic log-rank tests for groups divided by the threshold

**Fig. 3:**
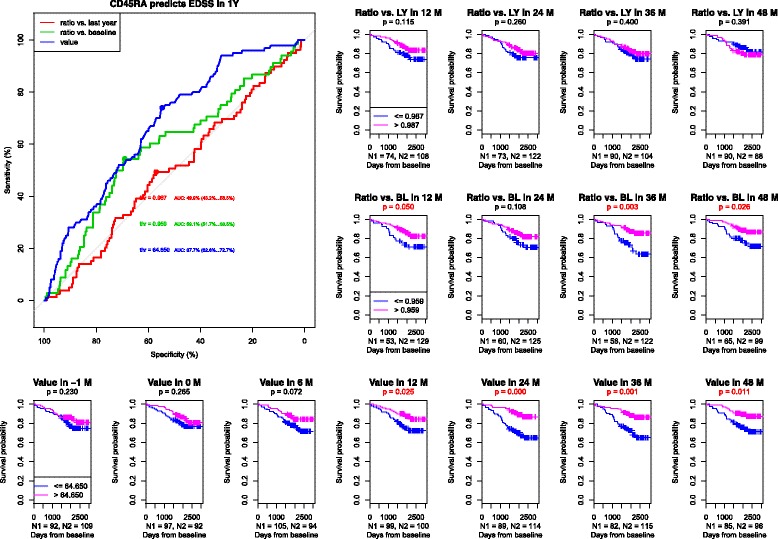
1) ROC curves of naive lymphocytes CD45RA+ to predict CDP at one year. AUC for the population relative value = 0.677, AUC for the population relative value change compared to baseline = 0.591, AUC for the population relative value change compared to a measurement one year before = 0.499. 2) Survival curves and asymptotic log-rank tests for groups divided by the threshold

**Fig. 4:**
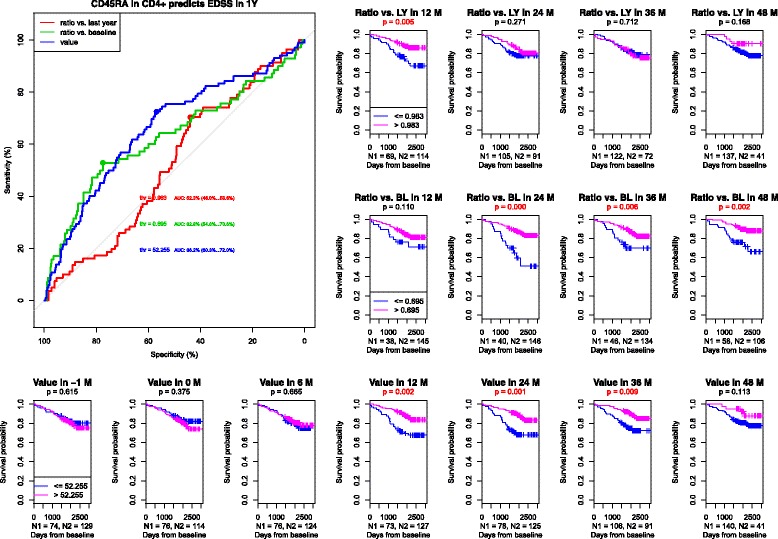
1) ROC curves of naive helper cells CD45RA+ in CD4+ to predict CDP at one year. AUC for the population relative value = 0.662, AUC for the population relative value change compared to baseline = 0.628, AUC for the population relative value change compared to a measurement one year before = 0.523. 2) Survival curves and asymptotic log-rank tests for groups divided by the threshold

We aimed to test the possibility of finding a threshold value that would distinguish patients with a higher or lower probability of relapse or CDP **(**the **“**value” in Figs. 1-4). More importantly, we aimed to measure changes in lymphocyte subpopulations longitudinally in each patient separately so that the disease course could be predicted. Therefore, we tested the ratio of current values versus values obtained a year before compared to baseline (Ratio vs. LY and Ratio vs. BL Figs. 1-4).

For the assessment of lymphocyte subpopulations, relative percentage is usually used, but absolute counts can be also calculated. As interferon beta decreases the overall lymphocyte count, including absolute values of respective subpopulations, we selected relative values for the statistical analysis. Since only results obtained before a relapse or CDP were statistically assessed, no other treatment than interferon beta could have influenced the parameters, and treatment was the same in all patients. The results obtained from patients after therapy escalation (18 natalizumab, 3 fingolimod) and also after changing for copaxone (6 patients) were excluded from this part of our study. Also methylprednisolone pulses were the same in all patients and the treatment was given at least 30 days before baseline**.**

No significant changes were observed in cytotoxic (CD8+) T lymphocytes, CD5+ cells and CD5+ B lymphocytes (CD19+) subpopulations as well as in activated T lymphocytes (DR+).

Only a few statistically significant changes in CD3+ T lymphocytes and CD4+ T lymphocytes were found in Kaplan-Meier estimators, while ROC curves did not show any clinically relevant predictive values of a new relapse or CDP.

The results assessing CD19+ cells, NK cells and naïve CD4+ T lymphocytes showed a much higher significance of these populations as a prediction of a clinical event.

#### B lymphocytes (CD19+)

In B lymphocytes, higher levels (the population relative value in our study was 9.5% of the total count of lymphocytes) were a positive predictive factor. (Figure1) The decrease of B lymphocytes below this level increased the probability of a relapse from month 6 until the end of the follow-up.

#### NK cells (CD3-CD16 + 56+)

Analysis of NK cells (Fig. 2) stressed the importance of long-term follow-up of representation of this population. The decrease of NK cells below 10.8% of the total count of lymphocytes increased the probability of CDP. Overall, a decreasing trend in the percentage of this population in peripheral blood was present, but transient more significant increase (of more than 28.7%) increased the probability of EDSS worsening in the first and third year of follow-up.

#### Naive cells (CD45RA+)

Percentages of naïve (CD45RA+) lymphocytes did not vary much (Table 1), but the statistical analysis revealed a significant relationship between this particular population and EDSS worsening. (Fig. 3) The probability of CDP was lower if the percentage of naïve lymphocytes was higher than 64.65% of total count of lymphocytes, and this was valid from the first year of follow-up. A more prominent decrease (to less than 95.9% of the baseline value) was a negative factor. However, the results were not significant when assessed with regard to the second relapse.

#### Naive helper cells (C45RA+ in CD4+)

Due to the fact that different subpopulations were included within CD45RA+ lymphocytes that may vary and influence the total results, we focused on one of the subpopulations – CD4+ naïve cells. (Fig. 4) In this subpopulation, the results were also statistically more significant when related to EDSS worsening. The percentage above 52.3% of total count of lymphocytes seemed to be important for protection against the clinical deterioration and the decrease of more than 30.5% as compared to the baseline value was a negative prognostic factor. No significant results were found in the context of the relapse.

The explanation for the fact that we found more significant changes in case of the CDP assessment than in the relapse one could be our incapability to guarantee examination at the same interval from relapse, as the examination was performed in predefined (yearly) intervals, irrespective of the presence and/or timing of relapse. We discovered CDP parameter to be more consistent for such an evaluation.

## Discussion

Various immunological markers with a potential of predicting conversion to CDMS or a relapse were not the only parameters analysed during a long-term follow-up of the CIS patients, which complemented results of radiological investigation of the same cohort. [6].

The results of our study corresponded with our long-time observation of immunophenotyping patterns in MS patients, i.e. higher level of CD4+ lymphocytes with a decreased expression of CD45RA molecules and lower proportion of B lymphocytes and NK cells. Almost all these results fit within normal values because of wide variability of these parameters. The same problem makes parametric statistical tests impossible for use in our and all such studies and this is the reason why we cannot use the value of these subsets as a MS diagnostic marker. We therefore decided to look at our data at an individual patient level and compared individual differences in observed subsets during 48 months of follow up in patients with newly diagnosed CIS treated with methylprednisolone at the beginning of the study and treated with interferon β either for the whole study (48 months) or until the time of a relapse or disability progression (before treatment escalation).

The key role in MS pathogenesis had been assigned to CD4+ cells for a long time and so this population was the most studied one, but low numbers of patients and different methods caused rather conflicting results [23]. In general, the levels of CD4+ cell population in MS patients (unless influenced by treatment (e.g. fingolimod)), are at the upper limit of normal range. Values of CD4+ T lymphocytes in our CIS patients at baseline were lower than we commonly found in MS patients in our laboratory and so we assumed significant augmentation in this subset during the disease progression to CDMS, but only a tendency to increase during the whole time of follow-up was found in all groups. This supports our anticipation, but we cannot designate whole CD4 + CD3+ lymphocyte subpopulation as a predictive factor for conversion to MS.

In 1986, *Mossman and Coffman* [24] presented the concept of distinct T helper cell subsets and MS was later considered a CD4+ Th1-mediated autoimmune disease [15] but not surprisingly the T-cell biology in vivo is more complex than a simple dichotomy. In addition to Th1/Th2, the Th17 subset and different classes of regulatory T cells (Treg) are involved in MS. Recent studies have shown that the T cells mediating MS can be heterogeneous, with Th17 cells predominating in some individuals and Th1 cells in others [25] The plasticity of different T cell subsets and emerging evidence that subset-signature cytokine expression is not as stable as initially believed [26] strongly support our attempt to find a more reliable marker as a predictor of conversion to CDMS.

In 1996 *Bomgioanni* [23] confirmed previous results [27] and showed a significantly decreased percentage of CD4 + CD45RA+ cells in peripheral blood during a relapse in RR-MS. Decrease in CD4+ CD45RA+ subset was also detectable one month before clinical relapse. [28] Up regulation of naive CD45RA+ T- lymphocytes and parallel down regulation of memory CD45RO+ cells seemed to be one of the main mechanisms by which Linomide inhibited the MS activity. [29] Also in this study, the higher levels of naive CD4+ cells seem to be a positive prognostic marker.

The above-mentioned studies prove the significance of monitoring naive CD4+ cell in the follow-up of our patients and our results are in agreement with them. Higher levels of naive CD4+ cells were shown to be a positive prognostic factor in our study, specifically more than 52.3% of naive cells in CD4+ cells and 64.6% of all naive lymphocytes. A reduction of naive helper cells by one third (exactly 30.5% in relationship to baseline) and only a slight reduction of total CD45RA+ lymphocytes (4.1% in relationship to baseline, statistically significant in the first, third and fourth year of follow-up) was associated with clinical worsening (CDP). According to these results, we could use monitoring of naïve CD4 + changes as an important predictive factor, but further studies will be required to examine the optimal frequency of (monitoring) measurement.

The contribution of B cells to the mechanisms of conversion to MS is shown not only by CSF B-cell associated biomarkers, but also by peripheral B cells. *Kreutzfelder* in 1992 [30] showed a reduction of CD19+ peripheral blood in MS patients. This observation combined with the reduced percentage of CD19+ circulating cells in patients with a stable RR form of MS *Bomgioanni* [23] suggested that such a decrease is due to the sequestration of cells within the CNS*. Lee-Chang* [31] supported this theory by finding an up-regulation of α4 integrin on peripheral B cells that may enhance B-cell accumulation within the CSF at the time of CIS.

Our results confirm reduction of B cells in PB as a negative predictive factor. The cut off level of 9.5% CD19+ cells of lymphocytes was calculated and a decrease below it increased the relapse probability. Overall augmentation of B cells during our study corresponded with the finding that circulating B cells became distinctly reallocated by long-term treatment with IFN-beta, which is compatible with the protective effects attributed to this drug [32]. Studies of the RR MS patients treated with IFN-1b confirmed that its in vivo therapeutic effects include: the inhibition of B cell stimulatory capacity and the B cells cytokine secretion changes, which may selectively inhibit Th17-mediated autoimmune response in RR MS. [33, 34] Given this, we could also conclude that lower levels of B cells in patients converting to CDMS were associated with lower responsiveness to interferon beta treatment and B cells analysis could predict need of treatment escalation so that relapse would be prevented.

Completely different results were found in one CD5+ B lymphocyte subpopulations. Earlier, this subpopulation was thought to be a potential source of autoantibodies, but its role in autoimmunity is still being investigated. Elevated CD5+ expression on peripheral B lymphocytes correlated with MS disease activity expressed by a number of gadolinium-enhancing lesions on MRI and inversely correlated with disease duration [35] and a recent study performed in blood of MS patients found an increase of CD5+ memory B cells in remitting stage of the disease [36]. *Villar* [20] concluded that increased percentages of blood CD5+ B cells were associated with further elevated risk of conversion to MS and relapse rate in these patients independently of cerebrospinal fluid oligoclonal bands presence and MRI. She calculated a cut off value of 3.5% of this subset for relapse risk. This value was based on mean +/− 2 SD of the percentage of blood CD5+ B cells of the control group. The association of blood CD5+ B cells with the conversion to MS suggests that this lymphocyte subset plays an important role in early phases of the disorder. We planned to use the same method for a cut off calculation, but, as we were looking for an individual predicted outcome, we used a different one.

Number of CD5 + CD19+ lymphocytes was generally lower in our study (Table 1b) and we were not able to confirm Vilar’s results neither by presented statistical calculation nor by former one [20]. We did not find significant changes in this population in relationship to the second relapse or CDP.

A recent phenotyping study that performed cytometric staining for multiple cell surface markers revealed lower frequencies of circulating CD8lowCD56 + CD3-CD4- cells in untreated patients with relapsing–remitting multiple sclerosis or clinically isolated demyelinating syndrome than in healthy controls [37]. The MS relapses and new brain lesions detected by magnetic resonance imaging are often preceded by a reduction in PB NK cell functional activity [38], but similar percentages of PB NK cells were detected in treated MS patients when compared to non-MS patients despite decreased frequency of CSF NK cells [39].

There was a slight gradual decrease of the NK cells in our study during the whole 4 year follow-up and the value of 10.8% could be considered as a critical point. Contrary to this, an increase at year one (and three) should predict CDP. This inconsistent result could be related to the changes in NK subpopulation: CD56bright NK cells. [40] During a follow-up we observed a decrease in the NK cells minor subpopulation, which lead to a lower control of T lymphocytes. Inter-annual increase of the NK cells preceding CDP could be caused by a transient increase of major CD56low population, which is found in chronic inflammatory process.

Nevertheless, in our study, we only assessed the total population of CD3-CD16 + 56+ NK cells as the representation of CD56bright NK cells was low in our patients, and we therefore did not consider this population suitable for a long-term assessment and statistical analysis. However, in recent years, some publications described the necessity of examination of this subpopulation.

Daclizumab-induced increased frequencies of circulating CD56bright NK cells were related to the therapeutic benefit of the drug [40]. Likewise, during therapy with IFNb, the total number of circulating natural killer cell declined slightly [41] or did not change [42] but there was a marked increase in the proportion of CD56high NK cells in both 12 month follow up studies. One of the possible mechanisms of action of IFN-β in MS is the enhancement of NK activity, especially CD56bright NK cells [38]. Given this finding, the analysis of NK cells seems to be a good predictive factor, in particular in combination with CD56bright subset analysis.

## Conclusions

In conclusion, our results confirm the potential role of monitoring early changes in immunophenotyping of peripheral blood lymphocytes for the prediction of a relapse and disability progression in patients after a first demyelinating event suggestive of MS. According to our results we can conclude that some changes in selected subpopulations should be considered as a signal for more frequent clinical and laboratory monitoring of our patients. More specifically, a decrease in B cells and NK cells populations (or a significant increase) and a marked reduction of naive CD4+ helper cells were the best predictors of a relapse or disability progression. Their predictive role has to be investigated and confirmed in further prospective studies. This study also shows difficulties of statistical assessment, and points to the fact that examination of inter-individual comparison of the measured parameters may be necessary instead of the comparison of whole groups of patients.

## Abbreviations

CDMS: Clinically definite MS
CIS: Clinically isolated syndrome
CNS: Central nervous system
CSF: Cerebrospinal fluid
DMTs: Disease modifying treatments
EDSS: Expanded Disability Status Scale
EDTA: Ethylenediamine tetra-acetic acid
IFNb: Interferon beta
IL: Interleukin
MoAb: Monoclonal antibody
MRI: Magnetic resonance imaging
MS: Multiple sclerosis
NK: Natural killer
OCBs: Oligoclonal bands
PB: Peripheral blood
TCR: T cell receptor
TLR: Toll like receptor
Treg: Regulatory T cells
